# Metabolite Profiling of Hydroponic Lettuce Roots Affected by Nutrient Solution Flow: Insights from Comprehensive Analysis Using Widely Targeted Metabolomics and MALDI Mass Spectrometry Imaging Approaches

**DOI:** 10.3390/ijms251810155

**Published:** 2024-09-21

**Authors:** Bateer Baiyin, Yue Xiang, Yang Shao, Jung Eek Son, Kotaro Tagawa, Satoshi Yamada, Mina Yamada, Qichang Yang

**Affiliations:** 1 Research Center for Smart Horticulture Engineering, Institute of Urban Agriculture, Chinese Academy of Agricultural Sciences, Chengdu National Agricultural Science & Technology Center, Chengdu 610213, China; bater@caas.cn (B.B.); xiangyue@caas.cn (Y.X.); 2 National Key Laboratory of Crop Genetic Improvement, College of Plant Science & Technology, Huazhong Agricultural University, Wuhan 430070, China; barnett@mail.hzau.edu.cn; 3 Department of Agriculture, Forestry and Bioresources, Seoul National University, Seoul 08826, Republic of Korea; sjeenv@snu.ac.kr; 4 Faculty of Agriculture, Tottori University, Tottori 680-8553, Japan; tagawa@tottori-u.ac.jp (K.T.); syamada@tottori-u.ac.jp (S.Y.); myamada.mimosa@gmail.com (M.Y.)

**Keywords:** MALDI-MSI, nutrient solution flow, hydroponics, metabolomics, root morphology, urban agriculture, plant factory

## Abstract

Root morphology, an important determinant of nutrient absorption and plant growth, can adapt to various growth environments to promote survival. Solution flow under hydroponic conditions provides a mechanical stimulus, triggering adaptive biological responses, including altered root morphology and enhanced root growth and surface area to facilitate nutrient absorption. To clarify these mechanisms, we applied untargeted metabolomics technology, detecting 1737 substances in lettuce root samples under different flow rates, including 17 common differential metabolites. The abscisic acid metabolic pathway product dihydrophaseic acid and the amino and nucleotide sugar metabolism factor N-acetyl-d-mannosamine suggest that nutrient solution flow rate affects root organic acid and sugar metabolism to regulate root growth. Spatial metabolomics analysis of the most stressed root bases revealed significantly enriched Kyoto Encyclopedia of Genes and Genomes pathways: “biosynthesis of cofactors” and “amino sugar and nucleotide sugar metabolism”. Colocalization analysis of pathway metabolites revealed a flow-dependent spatial distribution, with higher flavin mononucleotide, adenosine-5′-diphosphate, hydrogenobyrinic acid, and D-glucosamine 6-phosphate under flow conditions, the latter two showing downstream-side enrichment. In contrast, phosphoenolpyruvate, 1-phospho-alpha-D-galacturonic acid, 3-hydroxyanthranilic acid, and N-acetyl-D-galactosamine were more abundant under no-flow conditions, with the latter two concentrated on the upstream side. As metabolite distribution is associated with function, observing their spatial distribution in the basal roots will provide a more comprehensive understanding of how metabolites influence plant morphology and response to environmental changes than what is currently available in the literature.

## 1. Introduction

Unprecedented global population growth, rapid urbanization, and limited agricultural resources have exacerbated concerns regarding food security and sustainability. Moreover, traditional soil-based farming methods are increasingly challenged in meeting the increasing demand for fresh produce [1]. Hydroponic technology has emerged as a promising alternative, offering efficient water and land use concomitant through soilless cultivation methods. Nevertheless, several challenges hinder the widespread adoption and optimization of hydroponic agriculture [2], such as the limited understanding of the effects of nutrient solution flow dynamics on plant metabolism and growth. Although traditional soil-based farming practices have been extensively studied and optimized over centuries, hydroponic agriculture is relatively nascent and requires further research to maximize its potential [3].

The dynamic nature of nutrient solution flow in hydroponic systems poses challenges different from those of soil-based cultivation. Hydroponics involves growing plants in nutrient-rich water solutions, with the flow dynamics of these solutions playing a pivotal role in plant growth and metabolic processes [4]. In particular, the efficiency of nutrient absorption and plant growth is largely controlled by root morphology [5], including root length, branching patterns, and root hair density [6]. The adaptation of root structures to different growth environments constitutes an important strategy for plants to adapt to various natural habitats [7]. Under hydroponic conditions, plant roots are influenced by nutrient solution flow, which mechanically stimulates a series of biological responses, including gene expression, hormone synthesis, and signal transduction pathways, leading to changes in root growth and differentiation [8]. The increased root surface area associated with root growth can enhance the ability of plants to absorb nutrients and facilitate their adaptation to hydroponic environments [9]. Therefore, understanding the mechanisms regulating root morphology under mechanical stimulation is crucial for improving the growth efficiency and yield of hydroponically cultivated plants.

Plant morphology is closely associated with metabolite accumulation [10,11]. The growth and development of plant structures, such as roots, stems, and leaves, require substantial nutrient and energy support obtained via absorption from the growth medium and photosynthesis, respectively. These nutrients are metabolized within plants to produce various metabolites [12] that include a wide range of small organic molecules such as sugars, amino acids, organic acids, hormones, and secondary metabolites. These molecules not only participate in energy conversion and substance metabolism within plants but also play crucial roles in growth, development, response to environmental changes, and interactions with other organisms [13]. Moreover, some metabolites promote plant growth and development [14], whereas others inhibit plant morphological establishment [15]. Metabolites also participate in physical defense systems, influencing the plant’s interaction with the environment [16].

The controlled delivery of nutrients through hydroponic systems allows the precise manipulation of plant nutrition and metabolite accumulation, optimizing crop yield and quality [17]. However, the continuous circulation of nutrient solutions in hydroponic systems must be carefully managed to avoid adverse effects on plant health and productivity [18]. Understanding how different flow rates affect metabolic processes is, therefore, essential for optimizing hydroponic systems to maximize efficiency and yield. Accordingly, among the various factors influencing hydroponic cultivation, the dynamics of nutrient solution flow have garnered considerable attention because of their potential to modulate the plant root metabolism and overall growth efficiency [19].

Metabolomics provides a holistic approach to studying plant metabolism by simultaneously profiling and quantifying a wide range of small molecules (metabolites) involved in cellular processes [20]. Recent advancements in metabolomics, particularly comprehensive targeted and untargeted metabolomic techniques, have revolutionized the ability to investigate complex metabolic pathways affected by nutrient solution dynamics during hydroponic cultivation [21]. These technologies enable the comprehensive analysis of metabolite composition and dynamics, offering insights into how nutrient flow regulates metabolic pathways at different growth stages [21]. In particular, we previously identified 79 significantly different metabolites in lettuce roots cultivated under different flow conditions [21]. The identification of these differential metabolites facilitates the understanding of the specific effects of nutrient flow stimuli on root metabolism and the role of these metabolites in the response of plants to hydraulic mechanical stimuli. 

In this context, in this study, we aimed to leverage advanced metabolomic methodologies to comprehensively explore changes in metabolites within the root systems of hydroponically grown lettuce in response to nutrient solution flow dynamics. By elucidating these metabolic responses, we aimed to elucidate the underlying mechanisms by which nutrient flow influences plant root metabolism. Understanding these mechanisms is crucial to optimizing the design and management strategies of hydroponic systems to enhance the productivity and nutritional quality of lettuce and other leafy greens. We integrated cutting-edge metabolomic techniques, and our findings unravel the complexities by which nutrient solution flow influences the metabolic dynamics of lettuce roots in hydroponic systems, providing a theoretical foundation and practical guidelines for the future development of hydroponic agriculture.

## 2. Results

### 2.1. Wide-Target Metabolism

We used extensive targeted metabolomic techniques to detect metabolites in lettuce root samples subjected to four nutrient flow rates: 0 (F0), 7 (F7), 14 (F14), and 28 L/min (F28). We detected 1737 metabolites in the four treatment groups, including 214 amino acids and their derivatives, 288 phenolic acids, 76 nucleotides and their derivatives, 183 flavonoids, 11 quinones, 94 lignans and coumarins, 145 alkaloids, 180 terpenes, 123 organic acids, 205 lipids, 2 tannins, and 216 other substances.

**Figure 1:**
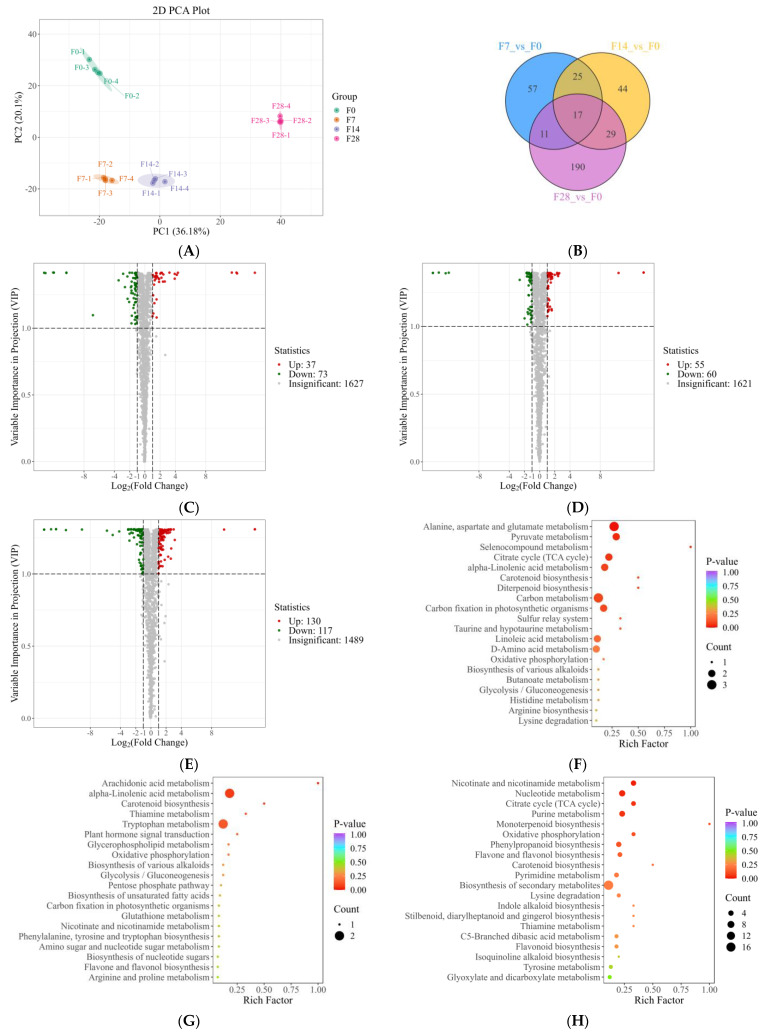
Wide-target metabolism of roots grown under different flow rates. (**A**) PCA of metabolism of roots grown under different flow rates; (**B**) Veen diagram of three comparison groups; (**C**) volcano plot of F7 vs. F0; (**D**) volcano plot of F14 vs. F0; (**E**) volcano plot of F28 vs. F0; (**F**) KEGG enrichment of F7 vs. F0; (**G**) KEGG enrichment of F14 vs. F0; (**H**) KEGG enrichment of F28 vs. F0.

The results of principal component analysis (PCA) (Figure 1A) showed a significant separation among the four treatment groups, indicating distinct metabolic profiles in each group. PC1 explained 36.18% of the variance in the original dataset, and PC2 explained 20.1%. The differential metabolite screening results revealed 17 common differential metabolites in the comparisons of F7 vs. F0, F14 vs. F0, and F28 vs. F0 (Figure 1B). The F7 vs. F0 comparison identified 37 up-regulated differentially expressed metabolites (DEMs) and 73 down-regulated DEMs (Figure 1C); in the F14 vs. F0 comparison, 55 up-regulated and 60 down-regulated DEMs were identified (Figure 1D); the F28 vs. F0 comparison identified 130 up-regulated and 117 down-regulated DEMs (Figure 1E). These results indicated that with increasing nutrient solution flow rate, the number of DEMs increased across all comparison groups.

The results of the DEM Kyoto Encyclopedia of Genes and Genomes (KEGG) (https://www.kegg.jp/, accessed on 9 January 2024) enrichment analysis showed that significantly enriched pathways for differentially expressed genes (DEGs) in the F7 vs. F0 comparison included “alanine, aspartate and glutamate metabolism”, “pyruvate metabolism”, “alpha-linolenic acid metabolism”, and “citrate cycle” (Figure 1F); in F14 vs. F0, these were “alpha-linolenic acid metabolism” and “tryptophan metabolism” (Figure 1G); in F28 vs. F0, these included “nicotinate and nicotinamide metabolism”, “citrate cycle”, and “nucleotide metabolism” (Figure 1H).

### 2.2. Non-Target Spatial Metabolomics

**Figure 2:**
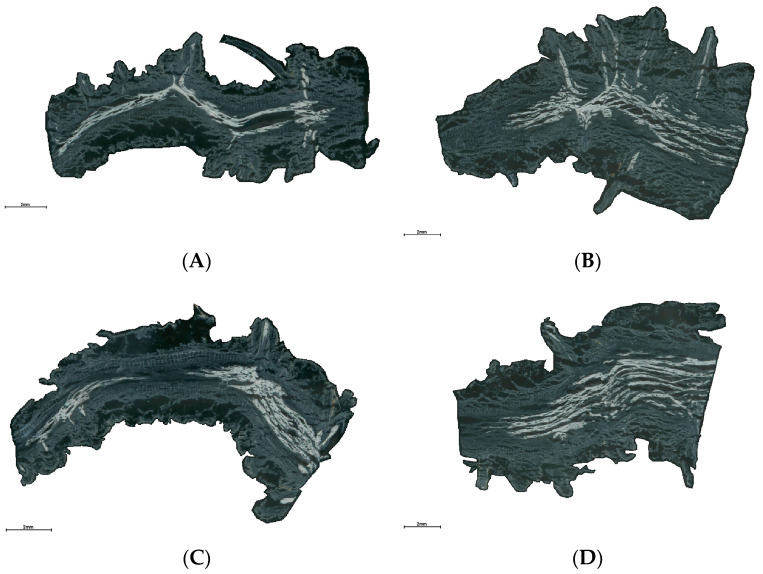
Scanned images of root base slices exposed to different flow rates. The upper side faces the water flow; the lower side faces away from the water flow. (**A**) Root grown under F0; (**B**) root grown under F7; (**C**) root grown under F14; (**D**) root grown under F28. The standard scale length in the figure is 2 mm.

**Figure 3:**
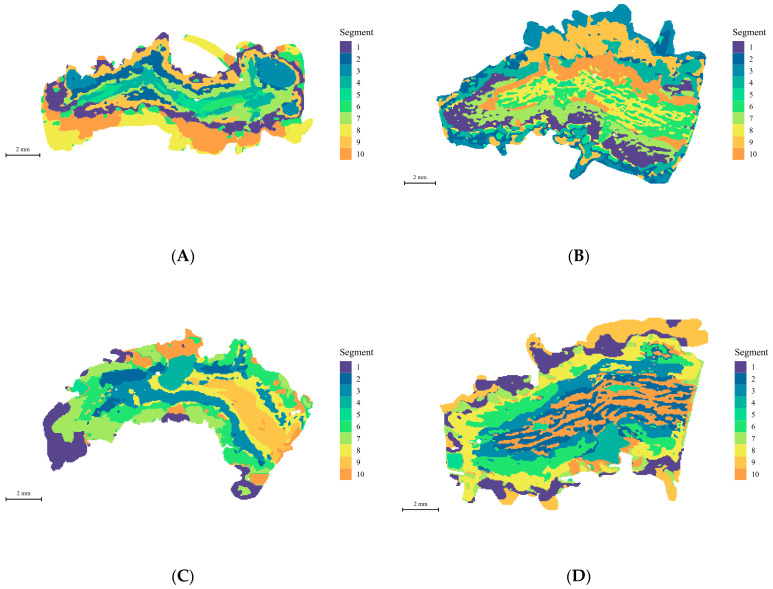
Spatial segmentation map of root base slices exposed to different flow rates. The different colors in the figure represent different regions of spatial segmentation. (**A**) Root grown under F0; (**B**) root grown under F7; (**C**) root grown under F14; (**D**) root grown under F28. The standard scale length in the figure is 2 mm.

**Figure 4:**
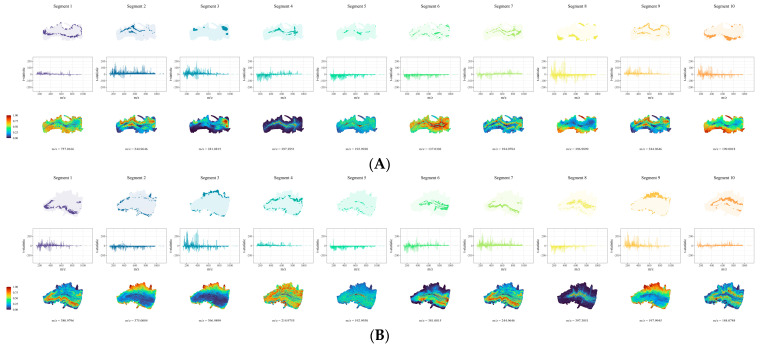

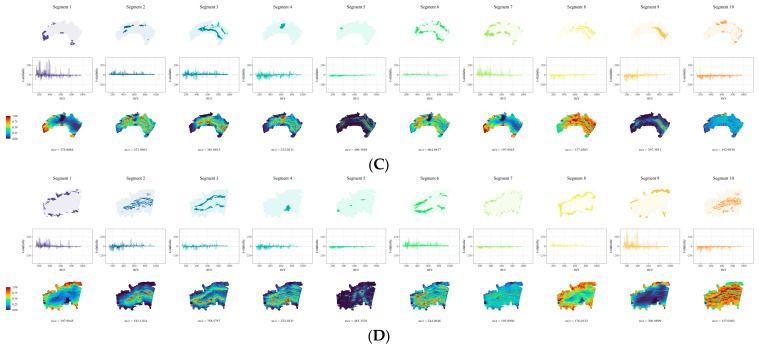
Spatial distribution map of metabolites of root base slices exposed to different flow rates along with quantity statistics and regional features. For each flow rate, the upper figure corresponds to spatial segmentation analysis of the respective regions; the middle figure shows quantity statistics for each target peak in the corresponding regions, with *m*/*z* on the horizontal axis and the t-statistic on the vertical axis; and the lower figure depicts spatial distribution maps of metabolites corresponding to regions with maximum statistical quantities. (**A**) Root grown under F0; (**B**) root grown under F7; (**C**) root grown under F14; (**D**) root grown under F28. The *m*/*z* is the “mass-to-charge ratio”, the ratio of the number of protons to the number of charges.

To investigate the spatial distribution of metabolites in the root base of lettuce under different nutrient solution flow rates, we employed non-targeted spatial mass spectrometry imaging techniques to analyze root samples. Scanned images of the root sections revealed marked differences in the curvature of roots between the different nutrient flow rate treatments (Figure 2). Using spatially aware nearest shrunken centroid clustering analysis, we obtained the partition information of metabolites in root samples under different flow rate treatments, with pixels within the same region exhibiting consistent metabolic trends. As shown in Figure 3, the root samples under the four different flow rate treatments were divided into 10 metabolite distribution regions. Subsequently, based on the results of spatially aware centroid analysis, the t-statistics of each target peak in each region were used for feature metabolite selection across the 10 regions of each group, with the metabolite with the highest t-statistic identified as the characteristic metabolite for that region (Figure 4). Detailed information on the characteristic metabolites in each region corresponding to each treatment is provided in Table S1.

**Figure 5:**
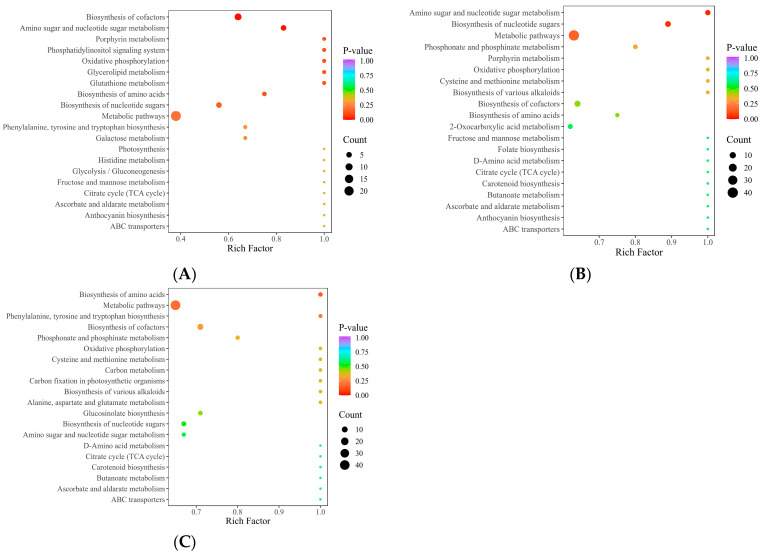
Kyoto Encyclopedia of Genes and Genomes (KEGG) pathway enrichment plots of differentially expressed metabolites (DEMs) in roots exposed to various flow rates. (**A**) F7 vs. F0; (**B**) F14 vs. F0; (**C**) F28 vs. F0.

We used fold-change (FC) ≥ 1.2 and FC ≤ 0.83 as criteria for the DEM screening. In the comparisons of F7 vs. F0, F14 vs. F0, and F28 vs. F0, we identified 710, 625, and 727 DEMs, respectively. KEGG enrichment analysis of the DEMs showed that in F7 vs. F0, pathways such as “biosynthesis of cofactors”, “amino sugar and nucleotide sugar metabolism”, and “biosynthesis of amino acids” were significantly enriched (Figure 5A). In F14 vs. F0, enriched pathways included “amino sugar and nucleotide sugar metabolism”, “biosynthesis of nucleotide sugars”, and “biosynthesis of cofactors” (Figure 5B). In F28 vs. F0, enriched pathways were “biosynthesis of amino acids”, “phosphonate and phosphinate metabolism”, “biosynthesis of cofactors”, and “amino sugar and nucleotide sugar metabolism” (Figure 5C).

**Figure 6:**
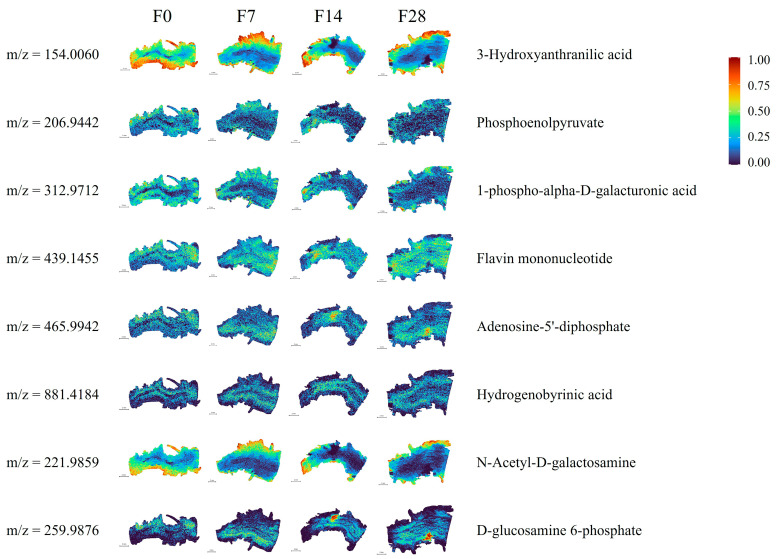
Common DEMs colocalization analysis spatial distribution map for roots exposed to various flow rates. The figure shows the spatial distribution of the eight common DEMs. Different colors represent the relative intensity of the substance in the area. Metabolite content increases from 0 to 1, as shown in the legend. *m*/*z* is the “mass-to-charge ratio”, the ratio of the number of protons to the number of charges.

Notably, the “biosynthesis of cofactors” and “amino sugar and nucleotide sugar metabolism” pathways were enriched in all three comparison groups, encompassing eight common DEMs among the two pathways and three comparison groups: 3-hydroxyanthranilic acid, phosphoenolpyruvate, 1-phospho-alpha-D-galacturonic acid, flavin mononucleotide, adenosine-5′-diphosphate (ADP), hydrogenobyrinic acid, N-acetyl-D-galactosamine, and D-glucosamine 6-phosphate. The spatial distributions and *m*/*z* values of the eight common DEMs are shown in Figure 6. Compared with their levels in the no-flow condition, in the presence of flow conditions, 3-hydroxyanthranilic acid, phosphoenolpyruvate, 1-phospho-alpha-D-galacturonic acid, and N-acetyl-D-galactosamine are down-regulated, and flavin mononucleotide, ADP, hydrogenobyrinic acid, and D-glucosamine 6-phosphate are up-regulated under flow conditions (Figure S1). 

**Figure 7:**
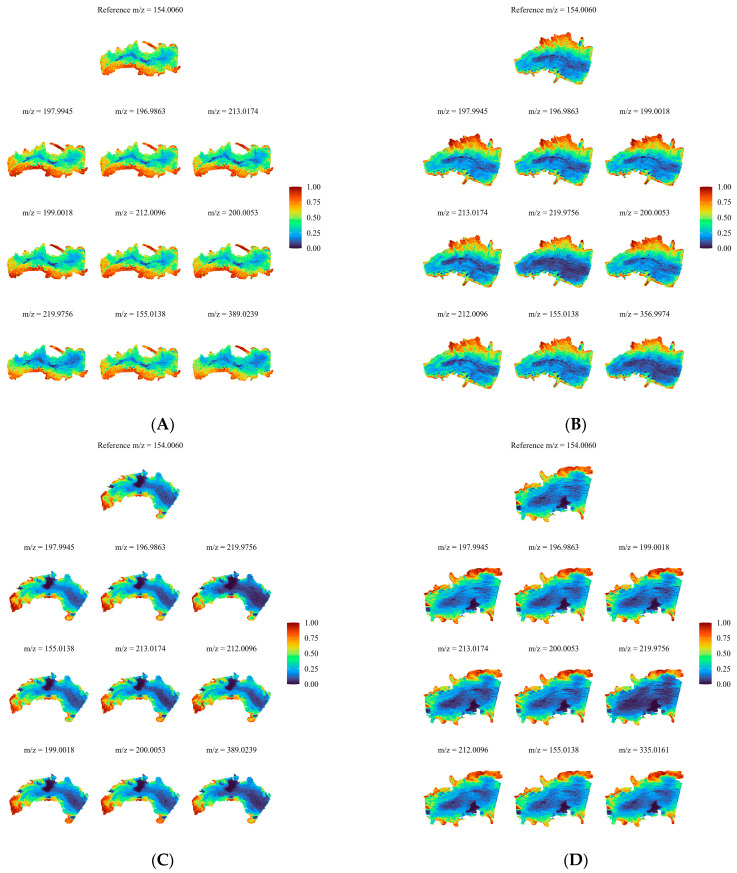
Metabolite colocalization analysis spatial distribution map for roots exposed to various flow rates. The figure shows the spatial distribution of the target metabolite and the top nine metabolites with Pearson correlation rankings. Different colors represent the relative intensity of the substance in the area. Metabolite content increases from 0 to 1, as shown in the legend. (**A**) Root grown under F0; (**B**) root grown under F7; (**C**) root grown under F14; (**D**) root grown under F28. *m*/*z* is the “mass-to-charge ratio”, the ratio of the number of protons to the number of charges.

We next conducted colocalization analysis of these eight common DEMs, calculating Pearson’s and Manders’ colocalization coefficients between these eight and the other metabolites. The aim of metabolite colocalization analysis is to identify metabolites whose spatial distribution patterns are consistent with those of the target metabolite and to explore the relationships between metabolites. Figure 7 shows the spatial distribution analysis of 3-hydroxyanthranilic acid under the different treatments. 3-Hydroxyanthranilic acid is mainly distributed in the surface layer of the roots, and its content after F0 treatment is significantly higher than that induced by the other three treatments. The colocalization analysis results for the remaining seven common DEMs are shown in Figure S2.

## 3. Discussion

In this study, we evaluated root samples grown under different flow rate conditions using wide-target metabolomics technology and identified 1737 compounds, of which 17 were DEMs, including terpenoids (dihydrophaseic acid, reynosin glucoside, sonchuside A), quinones (4,5,8-trihydroxy-α-tetralone), alkaloids (2,4-dihydroxyquinoline, quinine), phenolic acids (sodium ferulate), flavonoids (quercetin-3-O-neohesperidoside, quercetin-3-O-glucoside-7-O-rhamnoside, genistein-8-C-apiosyl(1→6)glucoside), lignans and coumarins (4β-hydroxypaulownin, (−)-lariciresinol, (7S,8R)-dihydrodehydrodiconferyl alcohol), lipids (lysoPE 16:1), and others (senkyunolide A, furoaloesone, N-acetyl-D-mannosamine) (Figure S3).

Dihydrophaseic acid is a product of abscisic acid (ABA) metabolic pathways. The hydroxylation pathway mediated by P450-type monooxygenase (CYP707A), an ABA metabolic pathway, can convert ABA into phaseic acid and dihydrophaseic acid through hydroxylation and isomerization processes [22]. ABA, a key plant hormone and signaling molecule, regulates the entire life processes of plant growth and development including root growth and elongation [23], as well as participates in plant resistance to various biotic and abiotic stresses. Thus, our results suggest that nutrient solution flow regulates root growth by affecting ABA metabolism in the roots.

N-Acetyl-D-mannosamine is an important natural sugar with various biological activities [24]. Pathways involving N-acetyl-D-mannosamine in amino- and nucleotide sugar metabolism are crucial for maintaining life activities. The metabolism of amino and nucleotide sugars releases energy or provides raw materials for other metabolic pathways [25]. Amino sugars are major components of glycoproteins that play supportive and recognition roles in the extracellular matrix [26]. Thus, our findings suggest that changes in root morphology induced by nutrient flow are related to sugar metabolism. Increased levels of metabolites typically indicate changes in certain biological processes or metabolic pathways. For example, if the level of a metabolite rises, it may suggest that the enzymes or biological processes involved in those pathways have become more active. N-Acetyl-D-mannosamine, which plays a crucial role in amino sugar and nucleotide sugar metabolism, may show elevated levels if these pathways are more frequently engaged. Increased metabolites can also reflect changes in nutrient supply, particularly in pathways related to sugar metabolism. For instance, if the root morphology changes due to nutrient solution flow, it may affect the sugar metabolism within root cells, leading to an increase in specific metabolites. Additionally, the increase in metabolites may be an adaptive response to environmental changes or stress. Cells might produce more of certain metabolites to cope with challenges posed by the nutrient solution flow. Elevated metabolites can impact the organism’s functions, such as amino sugars being essential components of glycoproteins, which play crucial roles in supporting and recognizing extracellular matrix functions important for root morphology and function. Overall, increased metabolites often reflect changes in intracellular metabolic activity, which can have significant biological implications and affect the overall health and function of the organism.

Wide-target metabolomics can provide insights into the metabolic responses of plants to specific environmental conditions and their effects on plant morphology by analyzing the overall characteristics of all metabolites within the plant [27]. However, this technology is limited by its inability to reveal the specific spatial distribution of metabolites in biological tissues. Consequently, wide-target metabolomics is unable to fully elucidate the alteration of metabolite accumulation and distribution in key locations associated with plant morphology, such as within the root system, induced by environmental differences. To overcome these limitations, integrating techniques such as spatial metabolomics with wide-target metabolomics can provide more comprehensive data on the distribution of plant metabolites.

Spatial metabolomics is an emerging research field that encompasses single techniques along with various analytical methods, such as mass spectrometry imaging (MSI), tissue sectioning, and data processing and analysis [28]. The combination of these technologies allows the analysis of metabolite spatial distribution at the microscopic level, revealing the complex interactions of metabolites within cells and tissues [29].

Spatial metabolomics has specific applications in botany, deepening the understanding of various aspects of plant physiology and ecology. For example, analyses of metabolite distribution in different organs (e.g., roots, stems, and leaves) or developmental stages (e.g., seedlings and mature plants) revealed changes in metabolic networks during plant growth and development. This facilitates the understanding of the functions and interactions of each organ throughout the plant life cycle, as well as the accumulation and distribution of specific metabolites at different developmental stages [30]. Spatial metabolomics can reveal how different plant parts respond metabolically to environmental stresses (e.g., drought, high temperatures, and salinity), aiding in the identification of metabolic biomarkers or key pathways involved in stress adaptation, providing a theoretical basis for breeding stress-tolerant varieties [31]. Moreover, secondary metabolites accumulate in specific tissues and cells of many medicinal plants. Spatial metabolomics can reveal the spatial distribution of these medicinal components within plants along with their biosynthetic pathways, promoting the rational development, utilization, and optimized production of medicinal plants [32]. Furthermore, spatial metabolomics can be used to explore the metabolic interactions between plants and symbiotic (e.g., mycorrhizal fungi) or pathogenic microorganisms. This reveals how these interactions affect plant metabolic networks and plant growth and development, deepening the understanding of the mechanisms underlying plant interactions with other organisms [33]. Thus, spatial metabolomics not only aids in understanding complex metabolic networks and biological functions in plant biology but also provides crucial scientific support for improving crop quality, enhancing stress resistance, and effectively utilizing medicinal plants [34].

In this study, we utilized wide-spectrum metabolomics to analyze the root bases showing the most flow stress. KEGG analysis revealed that in the three comparison groups, pathways such as “biosynthesis of cofactors” and “amino sugar and nucleotide sugar metabolism” were significantly enriched in the plant roots. Further colocalization analysis of the relevant metabolites in these two pathways indicated the presence of spatial distribution differences in the metabolites at the root apex under the different flow treatments (Figure 6). Flavin mononucleotide, ADP, hydrogenobyrinic acid, and D-glucosamine 6-phosphate exhibited higher levels under flow conditions than under no-flow conditions. Moreover, ADP and D-glucosamine 6-phosphate showed significantly higher concentrations on the downstream side than on the upstream side of the roots. ADP is the product of ATP hydrolysis or removal of a phosphate group by ATPase enzymes in biological organisms. ADP and ATP can be interconverted within cells to store and release energy, ensuring an energy supply for various life processes [35]. We speculate that under hydroponic cultivation conditions, the concentration of ADP within plant roots may affect plant growth and metabolic processes. An increase in ADP typically indicates a higher energy demand in the roots or a disruption in ATP synthesis, which could lead to changes in root growth and branching patterns. Properly regulating the flow of nutrient solution can optimize the metabolic balance of the roots, improving plant growth efficiency and adaptability. D-Glucosamine 6-phosphate is a precursor molecule for the synthesis of amino sugars and peptidoglycans, which are important components of cell walls. Therefore, D-glucosamine 6-phosphate plays a crucial role in maintaining cell wall structure and function [36]. Additionally, D-glucosamine 6-phosphate is involved in glucose metabolic pathways, where it is ultimately converted to glucose through a series of reactions, providing energy for the organism [37].

Under no-flow conditions, the levels of 3-hydroxyanthranilic acid, phosphoenolpyruvate, 1-phospho-alpha-D-galacturonic acid, and N-acetyl-D-galactosamine were higher than those under flow-treated conditions. Specifically, 3-hydroxyanthranilic acid and N-acetyl-D-galactosamine were significantly more highly concentrated on the upstream surface than on the downstream surface. The elevated concentration of 3-hydroxyanthranilic acid may lead to increased oxidative stress within the cells, affecting antioxidant capacity and inflammatory responses, potentially causing cellular damage. An increase in N-acetyl-D-galactosamine concentration could interfere with cell adhesion and signaling, impacting cell interactions and tissue repair. The rise in phosphoenolpyruvate might reflect enhanced glycolytic activity, thereby affecting cellular energy metabolism. High levels of 1-phospho-alpha-D-galacturonic acid may disrupt cell wall synthesis and stability, influencing cell structure and function. These changes in metabolite concentrations reveal the metabolic state and adaptive mechanisms of cells under different flow conditions, which is important for cellular biology research.

Phosphoenolpyruvate, a commonly observed biomolecule in cells, serves as a precursor of various organic acids, sugars, lipids, and secondary metabolites. It plays a crucial role in many metabolic pathways, such as gluconeogenesis/glycolysis, the citric acid cycle, and amino acid metabolism [38]. In the final step of glycolysis, phosphoenolpyruvate is catalyzed by pyruvate kinase, which transfers its phosphate group to ADP, generating ATP and pyruvate. This process releases a large amount of energy to support plant growth and development [39].

The distribution of these metabolites should be closely related to their functions. However, a limitation of this study is that only a single developmental period was observed and analyzed. Further observations and analyses of the spatial distribution of root metabolites across multiple growth stages would facilitate a comprehensive understanding of the influence of metabolites on plant morphology and their responses to environmental changes.

## 4. Materials and Methods

### 4.1. Cultivation

The experimental plant used in this study was lettuce (*Lactuca sativa* L. var. *ramosa* Hort.). The seeds were sown in seedling trays filled with moist vermiculite for germination. On the 7th day after sowing, the seedlings were transplanted into plastic containers filled with a standard nutrient solution at a consistent concentration and cultured for seven days. Seedlings with uniform growth were then selected and transplanted into cultivation tanks. The composition and concentration of the standard nutrient solution and cultivation conditions were as described in our previous study [8]. The experiment included four nutrient solution flow rate treatments, 0 (F0), 7 (F7), 14 (F14), and 28 L/min (F28), with four replicates containing five plants each per treatment. The pH of the nutrient solution in the cultivation tanks was 5.9, and the electrical conductivity was 2.28.

### 4.2. Reagents and Root Samples

Acetonitrile (liquid chromatography–mass spectrometry [LC–MS] grade) and methanol (LC–MS grade) were purchased from Merck (Darmstadt, Germany). Formic acid (LC–MS grade), trifluoroacetic acid (LC–MS grade), and 2,5-dihydroxybenzoic acid were purchased from Sigma (St. Louis, MO, USA). On the 14th day after planting, root samples were collected from all four treatment groups. The samples were immediately frozen in liquid nitrogen for 15 min and then stored at −80 °C. Among the collected samples, the entire root system of some lettuce plants was used for comprehensive targeted metabolomic analysis, and the basal parts of the lettuce roots were used for spatial metabolomic analysis.

### 4.3. Wide-Targeted Metabolomics Assays

We used an ultra-performance LC–electrospray ionization-tandem MS system for the comprehensive targeted metabolomic analysis of root samples. Metabolite extraction and determination from the samples used the methods described in our previous report [21]. MS data were processed using Analyst 1.6.3. Unsupervised PCA was performed using the statistics function prcomp within R (www.r-project.org, accessed on 9 January 2024). The data were unit variance scaled before use in unsupervised PCA. The accumulation patterns of metabolites in different samples were determined using hierarchical cluster analysis. The ComplexHeatmap package in R was used to create a clustering heatmap. The KEGG Compound database (http://www.kegg.jp/kegg/compound/, accessed on 9 January 2024) was used to annotate the identified metabolites, and then the annotated metabolites were mapped to the KEGG PATHWAY database (http://www.kegg.jp/kegg/pathway.html, accessed on 9 January 2024). Pathways mapped to significantly regulated metabolites were assessed via metabolite set enrichment analysis, and their significance was determined using hypergeometric test *p* values. The screening criteria for DEMs between groups were variable importance in projection (VIP) > 1 and FC ≥ 2.0 or FC ≤ 0.5.

### 4.4. Nontargeted Spatial Metabolomics Detection and Analysis

#### 4.4.1. Root Base Sample Slicing and Matrix Spraying

**Figure 8:**
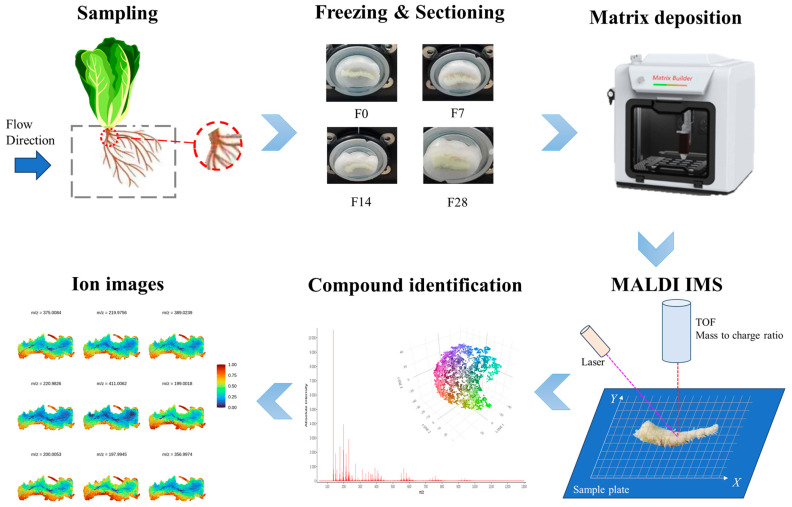
Matrix-assisted laser desorption/ionization–mass spectrometry imaging (MALDI-MSI) analysis of lettuce roots exposed to different nutrient solution flow rates. TOF: time of flight.

Under hydroponic conditions, fine roots sway with the nutrient solution flow; the force acting on them varies continuously with the degree of movement (fine roots may sway perpendicular or parallel to the flow). By contrast, the root base consistently bears the force of the flow (generally perpendicular to the flow direction). In this study, observations were conducted approximately 1–2 cm from the base of the root (Figure 8). Root base samples preserved at −80 °C were retrieved and equilibrated at −20 °C in a Leica CM1950 cryostat (Leica Microsystems, GmgH, Wetzlar, Germany) for 1 h. Subsequently, samples were affixed to the cryostat and sliced per the manufacturer’s instructions to a thickness of 30 μm. Sections were transferred onto pre-chilled indium tin oxide (ITO) slides and dried in a vacuum desiccator for 30 min. Once dried, a 15 mg/mL dihydroxybenzoic acid matrix solution in 90% acetonitrile was uniformly sprayed onto tissue-containing ITO slides using a TM-Sprayer matrix sprayer (HTX Technologies LLC, Chapel Hill, NC, USA). Instrument parameters were set as follows: temperature, 60 °C; flow rate, 0.12 mL/min; and pressure, 10 psi, with a total of 30 spray cycles and a 5 s drying time between each cycle.

#### 4.4.2. Matrix-Assisted Laser Desorption/Ionization–Time-of-Flight (MALDI–TOF) MSI Analysis

The root sections were analyzed using a prototype Bruker timsTOF flex MS system (Bruker Daltonics, Bremen, Germany) equipped with a 10 kHz smartbeam 3D laser. The laser power was fixed at 80% throughout the experiment. Mass spectra were acquired in positive mode. Mass spectral data were acquired over the mass range of *m*/*z* 50–1300 Da. The imaging spatial resolution was set to 50 μm for the tissue, with each spectrum consisting of 400 laser shots. MALDI mass spectra were normalized using the root mean square (RMS); the signal intensity in each image is shown as the normalized intensity. MS/MS fragmentations performed on the timsTOF flex MS system in the MS/MS mode were used for further detailed structural confirmation of the identified metabolites. Databases built by the Metware Corporation (Metware Corporation, Wuhan, China) and public databases were used to match the collected MS/MS spectra for substance identification.

#### 4.4.3. Data Analysis

The raw MSI data were imported into SCiLS Lab (Bruker Daltonics, Billerica, MA, USA) for smoothing and RMS normalization. This process provided the relative intensity information of different *m*/*z* values at each spatial point, which was converted into pixels on the imaging heatmaps. The entire root slice was selected as the region of interest. Metabolite identification was used to analyze the intensity data of all identified metabolites for spatially aware nearest shrunken centroid clustering to obtain partition information [40,41], in which pixels within the same region showed consistent metabolic trends. Spatially aware centroid analysis was used to calculate the t-statistics for each target peak in each region; region-specific metabolic features were selected based on the magnitude of the t-statistics. The identified metabolites were subjected to t-distributed stochastic neighbor embedding and uniform manifold approximation and projection analysis. Functional annotation of metabolites was performed using the KEGG database, followed by KEGG PATHWAY enrichment analysis based on DEM results (criteria for DEMs: FC ≥ 1.2 and FC ≤ 0.83 between comparison groups). Target metabolites selected from the differential analysis were subjected to metabolite colocalization analysis to identify metabolites with spatial distribution patterns consistent with the target metabolites; the aims were to uncover relationships between metabolites and to reveal the effect of environmental factors (nutrient flow) on the spatial distribution of metabolites.

## 5. Conclusions

This study explores the adaptive mechanisms of root morphology and metabolism in lettuce under the influence of nutrient solution flow rates in hydroponic conditions. Root morphology, which is crucial for nutrient uptake and plant growth, can adapt to various environmental conditions to enhance survival. When subjected to mechanical stimuli, such as solution flow, plants alter their root morphology and increase the surface area of their roots to improve nutrient absorption efficiency. Using metabolomics technology, we analyzed lettuce root samples under different flow rates and identified 1737 substances, including 17 differential metabolites. The findings highlight the role of dihydrophaseic acid, a product of the abscisic acid metabolic pathway, and N-acetyl-D-mannosamine, a factor in amino and nucleotide sugar metabolism, in regulating root growth through the influence of solution flow rate on organic acid and sugar metabolism. Spatial metabolomics revealed significantly enriched pathways in the root bases under stress, including “biosynthesis of cofactors” and “amino sugar and nucleotide sugar metabolism”, as defined by the KEGG. Colocalization analysis of pathway metabolites indicated a flow-dependent spatial distribution, with higher concentrations of metabolites such as flavin mononucleotide, adenosine-5′-diphosphate, hydrogenobyrinic acid, and D-glucosamine 6-phosphate under flow conditions, the latter two showing downstream-side enrichment. Conversely, in the absence of flow, metabolites like phosphoenolpyruvate, 1-phospho-alpha-D-galacturonic acid, 3-hydroxyanthranilic acid, and N-acetyl-D-galactosamine had higher concentrations, with the latter two concentrated on the upstream side. This study focuses on the relationship between metabolite distribution and root function, providing a deeper understanding of how metabolites influence plant morphology and response to environmental changes beyond the existing literature.

### Future Prospective of This Research Topic

Looking forward, our research aims to delve into several areas:

(a) The impact on diverse plant species: Applying similar metabolomics approaches to explore how different plant species respond to varying flow rates; identifying universal and specific adaptations.
(b) Long-term dynamics: Conducting a temporal study of lettuce in hydroponic systems to assess the temporal dynamics of root morphology and metabolites and their relationship with plant growth and yield; offering insights for optimizing growth environments and cultivation periods.
(c) The relationship of metabolites and gene expression: Integrating transcriptomic and genomic data to explore how specific metabolites regulate gene expression and influence root growth and adaptation; revealing deeper biological mechanisms.
(d) Multifactor environmental studies: Investigating the interaction effects of other environmental factors, such as temperature, light, nutrient concentration, and flow rates, on root metabolism and morphology to comprehensively understand their impact on plant growth.

In summary, future research will deepen our understanding of plant adaptation mechanisms in changing environments, providing a solid scientific basis for optimizing hydroponic technology, enhancing plant growth efficiency, and boosting agricultural production.

## Data Availability

All data generated or analyzed during this study are included in this published article.

## Supplementary Materials

The following supporting information can be downloaded at: https://www.mdpi.com/article/10.3390/ijms251810155/s1.
