# Supplementary material for: Metabolite Profiling of Hydroponic Lettuce Roots Affected by Nutrient Solution Flow: Insights from Comprehensive Analysis Using Widely Targeted Metabolomics and MALDI Mass Spectrometry Imaging Approaches
Source: Int J Mol Sci. 2024 Sep 21;25(18):10155. doi: 10.3390/ijms251810155 (PMC11432021; doi:10.3390/ijms251810155)
Supplement: Supplementary file 1 [file ijms-25-10155-s001.zip › Table S1. The characteristic metabolites information table of the corresponding region under different nutrient solution flow rate..pdf]

Table S1. The characteristic metabolites information table of the corresponding region under different nutrient solution flow rate.

Segment<br>Subject<br>Treatment		F0	F7	F14	F28
1	m/z	757.0166	380.9796	375.0084	197.9945
	compounds	26-O-Glucosyl- furost-25(27)-ene-3,22,26-triol-3-O- glucosyl(1→3)-galactoside(iso-1)	Diphyllin	Aminoimidazole carboxamide ribonucleotide	N-Acetylhistidine
2	m/z	244.9646	375.0084	252.9303	543.1364
	compounds	1,3,5-Trihydroxyxanthone	Aminoimidazole carboxamide ribonucleotide	7-Methoxyflavone	4-nitrophenyl 4-[bis(2H-1,3-benzodioxol-5-yl)(hydroxy)methyl] piperidine-1- carboxylate
3	m/z	381.0815	396.9899	381.0815	758.5757
	compounds	O-Sinapoylglucarolactone	Octose 1,8-bisphosphate	O-Sinapoylglucarolactone	Thioetheramide PC
4	m/z	397.3851	214.9755	222.0331	222.0331
	compounds	Stigmast-5-en-3-ol	2-(1h-1,2,4-triazol-5-yl)-1h-isoindole-1,3(2h)-dione	2-cyano-N-(4-sulfamoylphenyl) acetamide	2-cyano-N-(4-sulfamoylphenyl)acetamide
5	m/z	192.9930	192.9930	409.3859	465.3376
	compounds	5-Acetylthiophene-2-carboxylic acid	5-Acetylthiophene-2-carboxylic acid	6-aldehydo-ophiopogonone B	Adlupone
6	m/z	137.0303	381.0815	604.9437	244.9646
	compounds	3-Cyano-L-alanine	O-Sinapoylglucarolactone	N-Acetyl-L-phenylalanyl-3,5-diiodo-L-tyrosine	1,3,5-Trihydroxyxanthone
7	m/z	184.0784	244.9646	197.9945	192.9930

	compounds	Choline sulfate	1,3,5-Trihydroxyxanthone	N-Acetylhistidine	5-Acetylthiophene-2- carboxylic acid
8	m/z	396.9899	397.3851	137.0303	176.0132
	compounds	Octose 1,8-bisphosphate	Stigmast-5-en-3-ol	3-Cyano-L-alanine	2-Hydroxythiobenzamide
9	m/z	244.9646	197.9945	397.3851	396.9899
	compounds	1,3,5-Trihydroxyxanthone	N-Acetylhistidine	Stigmast-5-en-3-ol	Octose 1,8-bisphosphate
10	m/z	199.0018	184.0784	192.9930	137.0303
	compounds	Sphondin	Choline sulfate	5-Acetylthiophene-2-carboxylic acid	3-Cyano-L-alanine
